# Determinants of consistently high HIV prevalence in Indian Districts: A multi-level analysis

**DOI:** 10.1371/journal.pone.0216321

**Published:** 2019-05-07

**Authors:** Rajneesh Kumar Joshi, Sanjay M. Mehendale

**Affiliations:** 1 Symbiosis International University, Pune, India; 2 National AIDS Research Institute (ICMR), Pune, India; 3 Indian Council of Medical Research, New Delhi, India; Public Library of Science, UNITED KINGDOM

## Abstract

**Introduction:**

Factors associated with persistently high Human Immunodeficiency Virus (HIV) prevalence levels in several districts of India are not well understood. This study was undertaken to determine the association of socio-demographic characteristics, economic factors, awareness about HIV and Sexually Transmitted Infections (STIs), and condom use with consistently high HIV prevalence in the Indian districts and to ascertain whether these associations differed across various regions of India.

**Methods:**

This study was carried out including all 640 districts of India. Secondary analysis of data obtained from the Census of India-2011, HIV Sentinel Surveillance in India and District Level Household Survey-III was done. Population profile, socio-economic characteristics, levels of HIV/STI/condom awareness and condom use, were compared between the districts with and without consistently high HIV prevalence. Due to the presence of collinearity among predictor variables, we used principal component analysis and the principal component scores were included as covariates for further analysis. Considering the districts at level 1 and the regions at level 2, multi-level analysis was done by generalised linear mixed models. Variance partition coefficient and median odds ratio were also calculated.

**Results:**

Sixty-three districts with consistently high HIV prevalence were found clustered in the South and the North-east regions of India. Population size, density and urbanisation were found to be positively associated with consistently high HIV prevalence in these districts. Higher levels of literacy, better socio-economic status, higher proportion of population in reproductive age group and late marriages were positively associated with consistently high HIV prevalence in all regions of India except in the Southern region. Higher levels of knowledge about the role of condoms in HIV prevention and condom use were associated with low HIV prevalence at the district level.

**Conclusions:**

Considerable heterogeneity among factors associated with consistently high HIV prevalence at the district level in different regions of India necessitates special region-specific strategies for HIV control. Increasing awareness about HIV alone is not sufficient for controlling the HIV epidemic and there is a need to raise knowledge levels about preventive measures against HIV and promote the use of condoms amongst population.

## Introduction

In India, 2.14 (1.59–2.84) million people are estimated to be living with HIV infection [1]; the third largest number of People living with HIV/AIDS (PLHIV) in any country in the world. The national level HIV prevalence among adults in India, estimated to be 0.22% (0.16–0.30%) in year 2017, has shown a downward trend over the last few years. However, this downward trend at the national level masks the variations at the regional, state and district levels in the country [1, 2]. India's HIV epidemic is largely driven and maintained through contact between high-risk subpopulations like female sex workers (FSW), men who have sex with men (MSM), injecting drug users (IDU) and bridge populations with onward transmission to general population [3–5]. India launched National AIDS Control Program (NACP) in 1992 for the prevention and control of HIV/AIDS. This program has district level focus for implementation of prevention and control strategies, based on vulnerabilities and magnitude of HIV burden in a district [6, 7]. In spite of sustained efforts for HIV control for more than two decades, some districts in India are reporting consistently high HIV prevalence [1]. The factors associated with these substantial and unswerving epidemics of HIV in several pockets in India are not well understood.

The spread of HIV epidemic in a defined geographic region is known to be influenced by the interplay of socio-demographic, economic, cultural and behavioural factors [8–15]. Various studies conducted across the globe to understand the association of these factors with HIV have provided contrasting result [9, 11–14, 16–28]. Several studies have demonstrated the association of HIV with poverty while some studies reported higher HIV levels among person from better socio-economic strata [9, 13, 18–21, 25, 26, 29, 30]. Though HIV is considered to be associated with illiteracy, some studies have shown higher HIV prevalence among more educated groups [9, 11, 19, 20, 28, 31, 32].

Large variations in the results of these studies bring out the fact that the findings from one country or region of the world cannot be directly extrapolated to other countries, as the factors affecting dynamics of HIV spread vary with place and time [33]. There is a need to study complex inter-relationships between these socio-economic and behavioural factors with each other and with HIV to understand evolution and progress of HIV epidemic in a population. Joint United Nations Programme on HIV/AIDS (UNAIDS) has also advocated ‘Know your epidemic and your current response’ strategy to identify the key drivers of HIV epidemic, with focus on relationships between the epidemiology of HIV infection and the social conditions in the country [8].

Studies done in different parts of India had shown the association of lower literacy, higher urbanisation and socio-economic development with higher HIV prevalence levels [27, 28, 32, 34–37]. Other studies have also brought out low levels of HIV awareness and condom use in India [17, 38, 39]. However, most of these studies have been carried out in one or few states of India, and there has been no study at the national level to understand the relationships of socio-demographic factors with HIV in India. Hence, we planned this study to identify the districts with consistently high HIV prevalence in India and to determine whether various socio-demographic characteristics, economic factors, levels of awareness about HIV or sexually transmitted infections (STIs) and prevalent condom use at the population level are associated with consistently high HIV prevalence in these districts. We also assessed whether there were differences between association of these factors with consistently high HIV prevalence in the districts across various regions of India.

## Methods

### Study settings

India has 35 States and Union Territories, which are further subdivided into districts. A district is the basic unit of administration in India. We included all 640 districts of India (Census 2011) in this study. We considered 6 geographical regions of India- North, Central, West, South, East and North-East regions, for our region-specific analysis.

### Data sources

Secondary analysis of the data obtained from the following sources was carried out in this study:

#### HIV Sentinel Surveillance (HSS)

India has one of the largest HSS systems in the world. In India, HIV sero-prevalence surveys are carried out every two years over a period of three months among ante-natal clinic (ANC) attendees and high-risk groups (HRG) like FSW, MSM, IDU etc [40]. The methodology adopted in HSS is consecutive/random sampling with unlinked anonymous testing. The reporting unit level aggregate data from various HSS rounds conducted between 2007 and 2012 were obtained from National AIDS Control Organisation (NACO) for this study.

#### Census-2011

The Indian Census, conducted by the Government of India every 10 years uses extended de facto canvasser method and is the biggest single source of a variety of statistical information on different characteristics of the people of India [41]. In Census, every individual data is collected by visiting the households over a period of three weeks. We obtained district level data on various demographic variables from the latest census conducted in 2011 from the Office of the Registrar General and Census Commissioner of India.

#### District Level Household and Facility Survey (DLHS-3), 2007–08

The District Level Household and Facility Survey is a major demographic and health survey carried out in India, which provides information related to socio-economic characteristics, maternal and child health, contraception and reproductive health including knowledge about HIV/AIDS [42]. In DLHS-3, multistage stratified random sampling was used, in which primary sampling unit (village/urban wards) were selected from each strata using probability proportionate to size (PPS) sampling. In selected primary sampling units, required number of households were selected using systematic random sampling and ever-married women (age 15–49) and never married women (age 15–24) were interviewed.

### Outcome variable

#### Consistently high HIV prevalence in the district

NACP (India) considers districts reporting ≥ 1% HIV prevalence among pregnant women attending ANC clinics or ≥ 5% HIV prevalence among HRGs in HSS as the high HIV prevalence districts. For the present study, such districts reporting high level of HIV prevalence among ANC or HRG in each of the last three rounds of HSS (2007–12) were classified as consistently high HIV prevalence districts.

### Predictor variables

#### Population profile

The district level data on population size, population density, proportion of urban and tribal population, proportion of population in reproductive age (15–49 years) group, sex ratio (number of females /1000 males) and mean age of marriage were obtained from the Census of India 2011 and DLHS-III.

#### Socio-economic factors

DLHS-III data on the proportion of households in a district with low and high standard of living and the Census data on literacy rate were used.

#### HIV/STI awareness levels and condo m use

Data regarding the proportion of females in a district who had heard of HIV and STIs or reproductive tract infections (RTIs); who had knowledge about utility of condoms for prevention against HIV and who reported condom use for contraception were obtained from DLHS-III.

### Statistical analysis

The levels of above mentioned predictor variables in the study districts with and without consistently high HIV prevalence were compared using student’s unpaired t test. Sixty out of 63 districts with consistently high HIV prevalence and 532 out of 577 districts without consistently high HIV prevalence that had data on all predictor variables were selected for multivariable analysis. Collinearity between the predictor variables was assessed using correlation matrix and variance inflation factor [43].

#### Principal Component Analysis (PCA)

Principal Component Analysis was used due to the presence of collinearity among predictor variables, [44]. The data were checked for their suitability for the PCA by calculating Kaiser-Mayer-Olkin (KMO) index [45]. Kaiser’s criterion (Eigen value > 1) was used to ascertain the number of principal components (PC) to be retained in the final analysis. Varimax rotation was carried out on principal components retained and the PC scores for individual districts were calculated. Consistently high HIV prevalence in a district was considered as a binary outcome variable and the PC scores were used as covariates for further multi-level analysis.

#### Multi-level analysis

Clustering of the districts with consistently high HIV prevalence was found in certain regions of India, hence we carried out multi-level analysis as in case of clustered data the assumption of measured data being independent does not hold and can lead to correlated error terms and biased estimates of parameter [43, 46]. Accordingly, we considered two levels– 592 districts at the level 1 nested within six regions at the level 2, for generalized linear mixed model.

Firstly, a null or empty two level model, with only an intercept and region effects was fitted to ascertain the variance that existed between the regions. Then, the scores of four PC retained were added in the model to create a random intercept logit model. Subsequently, we extended the random intercept model to create a random slope logit model allowing both the intercept and coefficients of co-variates to vary randomly across the regions. In random slope model, we used likelihood ratio test to investigate whether the effect of a PC varied across the study regions [47].

Variance Partition Coefficients, indicating the proportion of total residual variance that is due to inter-cluster variation, were calculated by latent variable method [48]. Median Odds ratio, which depicts the median value of odds ratio between any two districts paired with the same covariates and chosen randomly from two different regions, was also calculated [48, 49]. R software -version 3.2.0 [50] was used for the statistical analysis.

### Ethics approval

This study has used site / district level aggregate data with no personal identifiers for the secondary analysis. The approval for this analysis was obtained from the Institutional Ethics Committee of National AIDS Research Institute, India.

## Results

**Fig 1:**
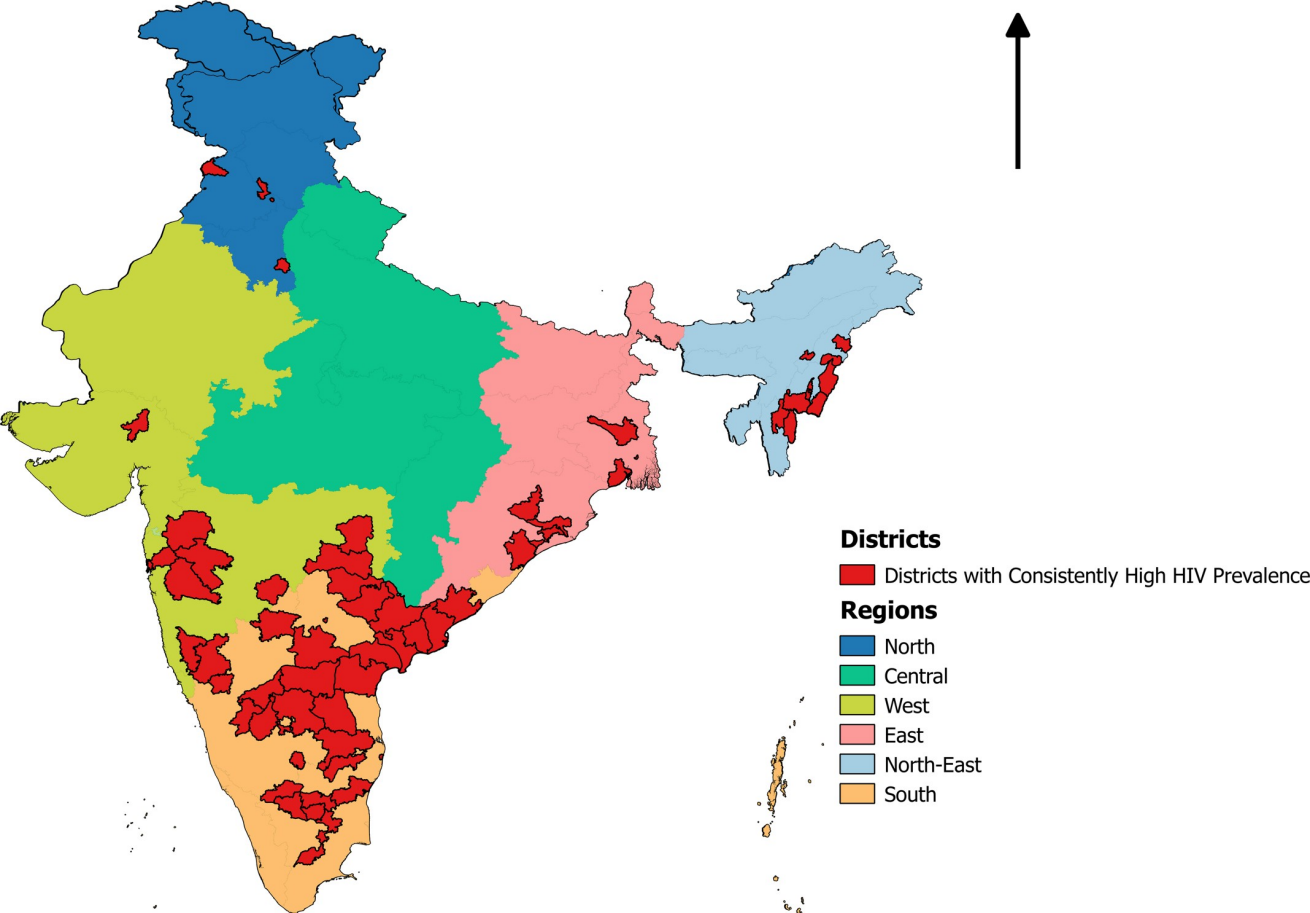
Map of India showing districts with consistently high HIV prevalence (2007–12). Districts with consistently high HIV prevalence are marked in red colour. These districts were found clustered in the South and the North-East regions of the country.

Sixty-three districts of the country (out of 640 districts) were found to have consistently high HIV prevalence (2007–12), as per the HSS data. The districts with consistently high HIV prevalence were found clustered in the South and the North-East regions of India (Fig 1).

There were significant differences between the districts with and without consistently high HIV prevalence with respect to population profile, economic variables and awareness about HIV/AIDS and condom use. (Table 1)

**Table 1:** Comparison of Population Profile (A), Socio-Economic Factors (B), Awareness about HIV/STIs and Condom Use (C) in the Districts with and without Consistently High HIV Prevalence in India: 2007–12.

Predictor Variables	Districts With Consistently High HIV Prevalence (Group I)	Districts Without Consistently High HIV Prevalence (Group II)
	Mean (SE)	Mean (SE)
Population size^a^, millions	3.21 (0.3)	1.75 (0.06)
Proportion (%) of urban population^b^	41.98 (3.34)	24.74 (0.82)
Proportion (%) of tribal population^a^	16.55 (3.69)	17.84 (1.11)
Population density^a^, population in hundreds /square km	30.26 (10.18)	7.72 (1.08)
Sex ratio^a^	959.45 (5.69)	943.91(2.58)
Age distribution of population^b^		
Proportion (%) below 15 years	26.43(0.48)	31.88 (0.24)
Proportion (%) 15–49 years	56.05 (0.29)	52.55(0.16)
Proportion (%) 50 years and above	17.14 (0.37)	15.31(0.14)
Age of marriage of males^c^, years	25.21 (0.21)	23.76 (0.10)
Age of marriage of females^c^, years	20.29 (0.22)	19.66 (0.08)
Literacy rate^a^		
Total (%)	76.94 (1.18)	71.80 (0.44)
Male (%)	83.26 (0.95)	80.07 (0.37)
Female (%)	70.44 (1.43)	63.01 (0.53)
Proportion (%) of households with low standard of living^d^	9.81 (1.27)	19.98 (0.79)
Proportion (%) of households with high standard of living^d^	30.06 (2.80)	20.45 (0.75)
Proportion (%) of females who have heard of HIV/AIDS^d^	87.19 (1.32)	64.19 (0.95)
Proportion (%) of females who have heard of RTI/STIs^d^	34.70 (1.60)	31.01 (0.73)
Proportion (%) of females who knew that consistent condom use can reduce the chances of getting HIV/AIDS^d^	26.93 (1.86)	35.71 (0.61)
Proportion (%) of females reporting use of condom as method of contraception^d^	3.79 (0.76)	4.96 (0.20)

Abbreviations: HIV, human immunodeficiency virus; STI, sexually transmitted infection

SE, standard error; km, kilometres; RTI, reproductive tract infection; AIDS, acquired immunodeficiency syndrome

^a^ 63 districts in group I and 577 districts in group II

^b^ 63 districts in group I and 576 districts in group II

^c^ 60 districts in group I and 533 districts in group II

^d^ 60 districts in group I and 542 districts in group II

**Fig 2:**
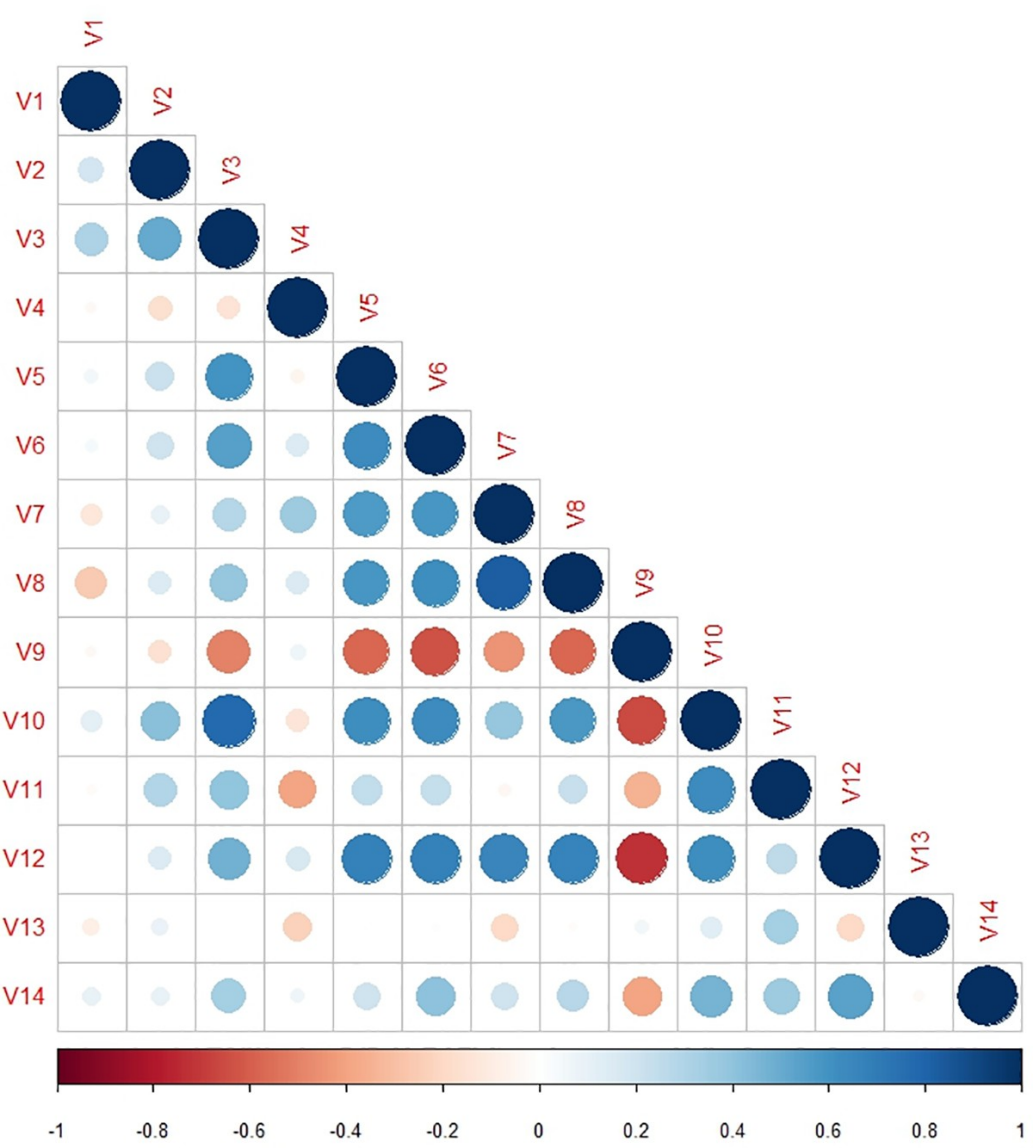
Correlation matrix of predictor variables for consistently high HIV prevalence at the district level in India. Size and colour density of circles are proportional to correlation between two variables. V1—Population size, V2- Population density, V3—Proportion of urban population, V4—Sex ratio, V5—Proportion population between 15–49 years, V6—Total literacy rate, V7—Mean age of marriage (males), V8- Mean age of marriage (females), V9 –Proportion of households with low standard of living, V10 –Proportion of households with high standard of living, V11—Use of condom as method of contraception, V12—Proportion of females heard of HIV/AIDS, V13—Proportion of women who knew that consistent condom use can reduce the chances of getting HIV/AIDS, V14—Proportion of females heard of RTI/STIs. (HIV, human immunodeficiency virus; AIDS, acquired immunodeficiency syndrome; RTI, reproductive tract infection; STI, sexually transmitted infection).

Correlation matrix (Fig 2) and variance inflation factors calculated revealed that there was high correlation between many predictor variables. Literacy was found to be positively correlated with HIV awareness levels (correlation coefficient (r) = 0.68, 95%CI: 0.63, 0.72) and STI awareness (r = 0.40, 95%CI: 0.33, 0.47); however, it did not have significant correlation with the knowledge about the role of condoms for HIV prevention among females (r = -0.01, 95%CI: -0.07, 0.09) at the district level.

### Principal Component Analysis (PCA)

KMO index calculated was 0.821, indicating suitability of the data for PCA. Four PC were retained for the final analysis based on Kaiser’s criteria. Cumulative variance explained by these four PC retained was 73%. The variables with significant loading (> 0.6) on these PC after varimax rotation are shown in Table 2.

**Table 2:** Principal component analysis with varimax rotation—significant loadings.

Predictor Variables	Principal Component 1	Principal Component 2	Principal Component 3	Principal Component 4
**(A) Population profile**				
Population size			0.78	
Proportion of urban population			0.66	
Population density			0.64	
Sex ratio		- 0.69		
Proportion of population between 15–49 years of age	0.78			
Mean age of marriage males	0.86			
Mean age of marriage females	0.91			
**(B) Socio-economic factors**				
Total Literacy rate	0.77			
Proportion of population with low standard of living	- 0.68			
Proportion of population with high standard of living	0.67			
**(C) HIV/STI awareness and condom use**				
Proportion of females who have heard of HIV/AIDS	0.78			
Proportion of females who have heard of RTI/STIs				0.86
Proportion of females who knew that consistent condom use can reduce the chances of getting HIV/AIDS		0.70		
Use of condom as method of contraception		0.74		

Abbreviations: HIV, human immunodeficiency virus; STI, sexually transmitted infection; AIDS, acquired immunodeficiency syndrome

RTI, reproductive tract infection

Principal component 1—Literate population in reproductive age group with better standard of living and late marriages

Principal component 2—Condom use and knowledge

Principal component 3 –Large population size with high density and urbanisation

Principal component 4- Awareness regarding RTI/STIs

### Multi-level analysis

#### Between region variance

Likelihood ratio test between single level null model without any explanatory variables and two level null model with the region as level 2 random effect was statistically significant (p < 0.01), depicting a significant variance between regions. Variance partition coefficient for the two level null model was 0.35 i.e. 35% of the residual variation in the propensity to report consistently high HIV prevalence in a district can be attributed to the unobserved region characteristics.

#### Random slope model

PC 1 had positive association with consistently high HIV prevalence in the districts in all the regions except in the Southern region. PC 3 had significant positive association while PC 2 had negative association with the outcome variable in all the regions of India (Tables 3 and 4). Variance Partition Co-efficient from this model was 0.244 and median odds ratio calculated was 2.66

**Table 3:** Multi-level analysis of association between principal components and consistently high HIV prevalence in Indian districts: 2007–12.

Generalised Mixed Effects Model			
**A *Fixed part of model***			
	**β**		**95% CI**
Intercept	- 3.20		-4.71, -2.10
PC 1	1.14		-0.26, 2.84
PC 2	- 0.68^b^		-1.14, - 0.29
PC 3	0.92^a^		0.62, 1.25
PC 4	0.19		-0.20, 0.58
**B *Region level -Random part of model***			
Intercept variance		1.06	
PC1 slope variance		1.95	
Variance partition coefficient		0.244	
Median Odds Ratio		2.66	

Abbreviations: HIV, human immunodeficiency virus; GEE, generalising estimating equations; CI, confidence interval; PC, principal component

β regression coefficient

a *P*< 0.001

b *P* < 0.05

**Table 4:** Region specific intercept and regression coefficient.

Regions of India	Intercept	β (PC1)	β (PC2)	β (PC3)	β (PC4)
North	- 2.94	0.78	- 0.68	0.92	0.19
Central	-4.09	2.33	- 0.68	0.92	0.19
West	-2.90	0.72	- 0.68	0.92	0.19
East	- 3.50	1.53	- 0.68	0.92	0.19
North East	- 4.01	2.24	- 0.68	0.92	0.19
South	-1.44	- 1.25	- 0.68	0.92	0.19

Abbreviations: PC, principal components

β regression coefficient

The direction of associations of the principal components and the individual variables with the districts reporting consistently high HIV prevalence is shown in Table 5. Single level model and Generalised Estimating Effect model, adjusted for region effect are also shown in S1 Table.

**Table 5:** Direction of association of predictor variables and principal components with consistently high HIV prevalence in Indian districts based on multi level analysis.

Predictor Variables	Principal Component on which Significant Loading	Direction of Association of Predictor Variable with PC	Direction of Association of PC with Consistently High HIV Prevalence in District	Direction of Association of Predictor Variable with Consistently High HIV Prevalence in District
**(A) Population profile**				
Population size	PC 3	Positive	Positive	Positive
Proportion of urban population	PC 3	Positive	Positive	Positive
Population density	PC 3	Positive	Positive	Positive
Sex ratio	PC 2	Negative	Negative	Positive
Proportion of population between 15- 49 years of age	PC 1	Positive	Positive^a^	Positive^a^
Mean age of marriage males	PC 1	Positive	Positive^a^	Positive^a^
Mean age of marriage females	PC 1	Positive	Positive^a^	Positive^a^
**(B) Socio-economic factors**				
Total literacy rate	PC 1	Positive	Positive^a^	Positive^a^
Population with low standard of living	PC 1	Negative	Positive^a^	Negative^a^
Population with high standard of living	PC 1	Positive	Positive^a^	Positive^a^
**(C) HIV/STI awareness and condom use**				
Proportion of female heard of HIV/AIDS	PC 1	Positive	Positive^a^	Positive^a^
Proportion of females heard of RTI/STI	PC 4	Positive	No significant association	No significant association
Proportion of females who knew that consistent condom use can reduce the chances of getting HIV/AIDS	PC 2	Positive	Negative	Negative
Use of condom as method of contraception	PC 2	Positive	Negative	Negative

Abbreviations: PC, principal component; HIV, human immunodeficiency virus; STI, sexually transmitted infection; AIDS, acquired immunodeficiency syndrome; RTI, reproductive tract infection

^a^ in all regions of India except in South India, where association is in opposite direction

## Discussion

### Profile of the consistently high HIV prevalence districts in India

Our analysis shows that the districts with a large population size, high population density, more urbanisation, higher proportion of population in the reproductive age group, higher sex ratio, better standard of living and higher mean marriage age were more likely to have consistently high HIV prevalence levels in India. We found that higher knowledge levels about the role of condoms for HIV prevention as well as the use of condoms at the population level were associated with lower HIV levels in the districts. Our findings are similar to other studies [32, 34] which demonstrated higher HIV levels in districts with higher urban population, better socio-economic conditions and lower condom use. The positive association of the awareness levels of HIV with consistently high HIV levels in a district in our study might be due to reverse causality. Better employment opportunities in big cities result in influx of predominantly young migrants leading to accumulation of HRG population in these districts [38, 51–53]. Separation from families, flourishing sex trade, marriages at a later age and availability of money from employment provide opportunities for sexual encounters with multiple partners–both commercial and non-commercial [35, 36, 52]. These conditions coupled with lack of information about HIV prevention and condoms might increase the risk of unsafe sex and chances of HIV acquisition [17, 39, 51]. Overcrowded urban areas also known to have concentrations of other HRGs like FSW, MSM etc., which can further fuel the HIV epidemic in these districts [4, 5, 54, 55]. Another factor which may lead to higher HIV prevalence in these economically developed districts is the better availability of antiretroviral treatment and other medical facilities, which can cause in-migration of HIV positive persons as well as better survival of HIV positive patients in these districts.

### Literacy, HIV awareness and HIV prevalence in districts

In our study, the districts with consistently high HIV levels had higher literacy rates as well as higher awareness levels about HIV, but lower levels of knowledge about utility of condoms for HIV prevention as compared to other districts of India. This analysis shows that higher levels of literacy and awareness about HIV/AIDS does not necessarily get translated to better knowledge about HIV prevention and low HIV prevalence at the population level. There is a significant gap between awareness on HIV and knowledge on HIV prevention methods in India which needs to be bridged. A study by R Ray et al [17] has also made similar observations regarding knowledge deprivation of HIV/AIDS in India.

### Heterogeneous HIV epidemic in India

Our study shows that the population level factors associated with HIV vary between different regions of the country. Factors like high literacy, better socio-economic status, higher proportion of population in reproductive age group and late marriages were positively associated with consistently high HIV prevalence among the districts in all regions of India except in the Southern region where the association was negative. Other studies carried out in the Southern India have also shown similar results [35, 37]. South India was the first and the worst HIV affected region of India in the initial phase of HIV epidemic. It might be possible that the HIV epidemic in South India has evolved over time and is not influenced by factors such as better socio-economic conditions etc. anymore, unlike other parts of India.

### Implications for HIV control in India

The results of this study help us not only in profiling the consistently high HIV prevalence districts in India in terms of socio-demographic and economic variables but also facilitate understanding of the association of population characteristics with HIV disease burden in India. This study has shown that better developed, urbanised districts with large population size, better socio-economic status of population are more likely to have consistently high HIV prevalence levels. Hence, the HIV control program needs to focus on these districts and keep in mind the possibility of emergence of HIV problem in districts which are showing signs of rapid urbanisation and socio-economic development. Special region-specific strategies for HIV control should be planned and implemented in India based on the principal drivers of HIV epidemic in different regions of India. We also recommend that Information, Education and Communication (IEC) component of HIV/AIDS Control Program of the country should focus not only on creating HIV awareness, but also on raising the knowledge levels about HIV prevention and role of condoms in that.

### Strengths and limitations of the study

We have analysed nationwide data and used population-based data sources for our study. Multi-level analysis inform us about the relationships of the population level factors with HIV prevalence at the national and regional levels, and also help us to understand the inter-regional variation in these associations. However, one needs to be aware of ‘ecological fallacy’ while interpreting the results of this study, as we have studied associations at the district level which may not necessarily hold true at an individual level. Another limitation in this study is that we could use awareness levels of HIV/STI/condom of only females for our analysis, since no survey in India has captured nationwide district level data of these variables for the male population. Information regarding district level distribution of variables specifically related to MSM, IDU is not available from any data source in India. Hence, the same could not be included in the study. We also had to exclude some districts from multivariable analysis as complete data on all the variables were not available for them. However, we assume that the exclusion of these districts from multi-variable analysis has not resulted in significant selection bias as the results from the multi-variable analysis are generally in the same direction as from the bivariable analysis.

## Conclusions

The population level factors are important determinants of HIV in India. HIV epidemic in India is associated with different factors in different regions. Population size, population density and urbanisation were positively associated with consistently high HIV prevalence in the Indian districts. Literacy, better socio-economic status and late marriages were found to be positively associated with consistently high HIV prevalence among the districts in most of the regions of India, however, in the Southern region these factors were negatively associated. Regular studies should be undertaken to better understand the associations of various socio-demographic factors with HIV, as the drivers of HIV epidemic change with place and time.

## Supporting information

S1 Table Single level and generalised estimating equation models of association between principal components and consistently high HIV prevalence in Indian districts (adjusted for region effect). (DOCX)
