# Supplementary material for: Determinants of consistently high HIV prevalence in Indian Districts: A multi-level analysis
Source: PLoS One. 2019 May 7;14(5):e0216321. doi: 10.1371/journal.pone.0216321 (PMC6504102; doi:10.1371/journal.pone.0216321)
Supplement: S1 Table — (DOCX) [file pone.0216321.s001.docx]

**S1 Table.** **Single Level and Generalised Estimating Equation Models of Association Between Principal Components and Consistently High HIV Prevalence in Indian Districts (Adjusted for Region Effect)**

|  | |  | **Single Level Analysis** | |  | **Generalised Estimating Equation** | | | |
| --- | --- | --- | --- | --- | --- | --- | --- | --- | --- |
|  | |  | **β** | **95% CI** |  | **β** | **95 % CI** | |  |
| Intercept |  | | - 3.45 | -5.30,  -2.00 |  | - 2.52 | -3.24,  -1.80 |  | |
| PC 1 |  | | 0.14 | - 0.29,  0.58 |  | 0.31 | -0.81,  1.43 |  | |
| PC 2 |  | | - 0.14 | -0.72,  0.43 |  | - 0.54^a^ | -0.81,  -0.27 |  | |
| PC 3 |  | | 0.91^a^ | 0.61,  1.24 |  | 0.84^a^ | 0.50,  1.18 |  | |
| PC 4 |  | | 0.17 | -0.20,  0.54 |  | 0.07 | -0.50,  0.65 |  | |

PC Principal Component

β regression coefficient

a *P*< 0.001
